# TA*p63 and GTAp63 achieve tighter transcriptional regulation in quality control by converting an inhibitory element into an additional transactivation domain

**DOI:** 10.1038/s41419-019-1936-z

**Published:** 2019-09-17

**Authors:** Susanne Pitzius, Christian Osterburg, Jakob Gebel, Georg Tascher, Birgit Schäfer, Huiqing Zhou, Christian Münch, Volker Dötsch

**Affiliations:** 1 Institute of Biophysical Chemistry and Center for Biomolecular Magnetic Resonance and Cluster of Excellence Macromolecular Complexes (CEF), Goethe University, Frankfurt/Main, Germany; 2 Institute of Biochemistry II, Faculty of Medicine, Goethe University, Frankfurt/Main, Germany; 3 Department of Human Genetics, Radboud University Medical Center, Nijmegen, The Netherlands; 4 Frankfurt Cancer Institute, Frankfurt/Main, Germany; 5 Cardio-Pulmonary Institute, Frankfurt/Main, Germany

**Keywords:** Biophysical chemistry, Tumour-suppressor proteins

## Abstract

The p53 homolog p63 plays important roles in development of epithelial tissues and quality control in germ cells. These two functions are executed by two distinct isoforms of p63. They are created by different promotors resulting in isoforms having either an N-terminal transactivation domain (TAp63) or a truncated form (ΔNp63). In addition to these two N-terminal isoforms a third one with an even longer N-terminus, named TA*p63, has been found. A fourth N-terminal isoform, GTAp63, that closely resembles TA*p63 was discovered in male germ cells where it is involved in genetic quality control. Here, we characterize TA*p63α and GTAp63α and show that their N-terminal extensions stabilize the closed and only dimeric conformation adopted by the shorter TAp63α protein. Both proteins can be activated by the two kinases Chk2 and CK1 resulting in the open tetrameric state. In this conformation, the N-terminal extension acts as an additional transactivation domain enhancing transcriptional activity. Through this mechanism, the difference in transcriptional activity between the repressed and the active state of the protein gets enhanced relative to TAp63α. Finally, we show by mass spectrometry that TA*p63α is expressed in the breast cancer cell line Sum159 at the protein level together with mutant p53. Upon doxorubicin treatment, TA*p63α gets activated, providing a potential new tool to fight cancer.

## Introduction

Sequencing of the human genome has identified ~23,000 open reading frames, which is significantly lower than the number of genes initially estimated and lower than the number found in other, more primitive organisms, e.g., *Daphnia pulex*, which has ~31,000 genes^1^. At the protein level, however, the complexity of the human proteome is enhanced by splicing events as well as the use of alternative promotors and translation start sites. These processes create isoforms with different activities. By tissue specific isoform expression the genome encoded protein functions are vastly expanded. One example for such an assignment of tissue specific functions to individual isoforms is the family of the tumor suppressor p53 with its three members p53, p63, and p73. Each of the three proteins exists in several isoforms created by the combination of different promotors producing various N-termini and C-terminal splicing events^2,3^. The tissue specific function of individual isoforms is best understood for p63. The ΔNp63α isoform is expressed in the basal layer of stratified epithelial tissues such as skin, where it is involved in regulation of proliferation and differentiation of keratinocytes^4,5^. It is characterized by the replacement of the first 69 amino acids (aa) of full length TAp63α with a 14 aa isoform unique peptide due to the use of an alternative promotor^2^. Inactivation of this isoform in a mouse model results in severe developmental defects such as limb truncations, lack of a multi-layered skin and other epithelial structures^4,5^.

The second p63 isoform for which a specific cellular function has been identified is TAp63α, which includes the full length N-terminal transactivation domain (TAD). This isoform is highly expressed in oocytes where it serves as a genetic quality control factor^6,7^. Mammalian oocytes get arrested in prophase of meiosis I in which they are stored in primordial follicles for extended periods of time. In resting oocytes, TAp63α adopts an inactive, closed and only dimeric conformation^8,9^. Upon detection of DNA double strand breaks it gets activated adopting an active, open and tetrameric conformation that triggers oocyte death via the expression of the two BH3-only proteins NOXA and PUMA^10,11^. This activation requires the consecutive action of the priming kinase Chk2^12^, phosphorylating a single serine in TAp63α and the executioner kinase CK1 which phosphorylates four more residues^13^.

During the initial cloning of p63 a third N-terminal variation, called TA*p63, was reported^2^. TA*p63 is created by yet another translation start site that adds 39 aa N-terminally to the TA domain. In transactivation (TA) assays in Saos2 cells on a p21 promotor construct TA*p63α is even more transcriptionally repressed than TAp63α^2^. Furthermore, it was found that in testis of humans and great apes an isoform is expressed that is N-terminally extended as well. This isoform named GTAp63 was created by the integration of the long terminal repeats (LTR) of the human endogenous retrovirus 9 (ERV9) about 15 million years ago^14^. GTAp63α is suggested to be the counterpart of TAp63α in male testes, responsible for insuring the genomic integrity. Upon DNA damage, the isoform can be activated via caspase cleavage in its C-terminus, which removes the transactivation inhibitory domain (TID)^15^. However, in the absence of genotoxic stress, GTAp63α is inactive, although it is highly and constitutively expressed in unstressed spermatogenic precursors. While the expression in spermatogenic precursors defines the tissue specific function for GTAp63α, no such biological function was so far reported for the TA* isoform. Recently, however, a mutation in the TA* specific domain of p63 was identified in a patient showing limb truncations, providing for the first time a link between this particular isoform and its biological function (will be reported in a separate manuscript). In addition, it has been reported that mutations in the TA* specific peptide occur in some cancer patients (https://cancer.sanger.ac.uk/cosmic, http://www.cbioportal.org/). Here we describe our investigations of the molecular mechanisms that regulate the transcriptional activity of TA*p63α and GTAp63α as the basis for understanding their specific function.

## Results

### The N-terminal elongation of TA*p63α and GTAp63α leads to a further stabilization of the kinetically trapped dimeric conformation

**Fig. 1:**
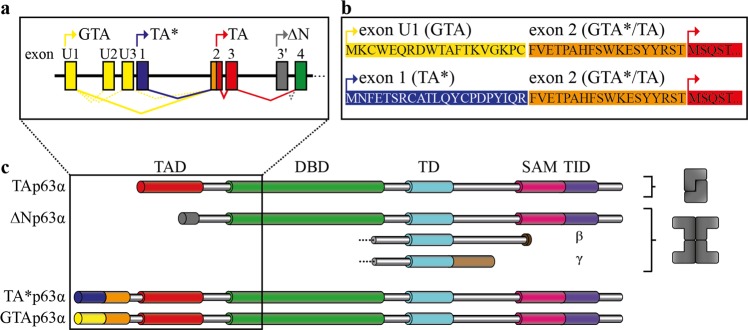
Exons encoding the N-terminal p63 isoforms, domain architecture as well as oligomeric conformation of p63 isoforms. **a** Testis specific GTAp63 is encoded by an upstream exon, named U1, originating from retroviral LTR insertion. mRNA splicing fuses these exons directly to the exon 2, on which the GTA*peptide and the N-terminal part of TAD are located, in this way skipping exon 1. TA*p63 is encoded on exon1. Main splicing events are illustrated by solid lines, less frequently splicing by dotted lines, start codons are indicated by arrows. **b** Amino acid sequences of the N-terminal p63 isoforms of the corresponding exons, start codons are indicated by arrows. **c** Domain architecture and oligomeric conformation of p63 isoforms. GTAp63 and TA*p63 possess an N-terminal elongation compared to TAp63, comprising the TA*- or GTA-peptide, respectively, and the common GTA*-peptide, whereas the residual domains are shared by all three isoforms. ΔNp63 possess a truncated transactivation domain. The C-terminal β- and γ-isoforms are created via alternatively splicing. Only the inactive state of TAp63α has a dimeric oligomeric conformation, all other TAp63 and ΔNp63 isoforms show a tetrameric conformation

The sequences defining the TA*p63 and GTAp63 specific N-termini can each be divided into two subdomains: While the C-terminal 18 aa of both peptides are identical (called in the following GTA* peptide) the N-terminal sequences diverge (called TA* and GTA peptides). Figure 1 provides an overview of the different p63 isoforms, the primary sequences of the TA*p63 and GTAp63 specific N-termini as well as the different transcription start sites.

**Fig. 2:**
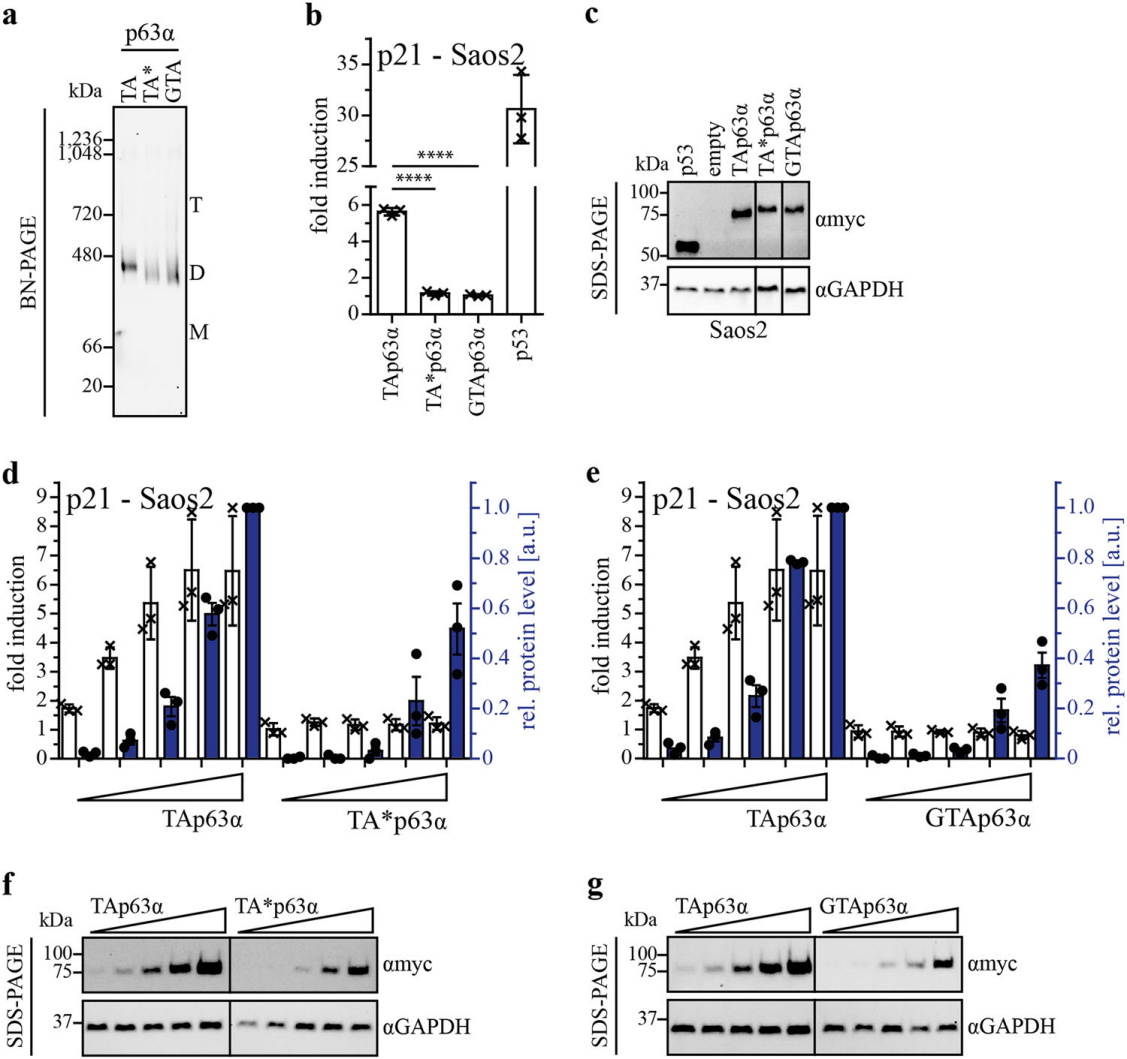
Oligomeric conformation and transactivation potential of TA*p63α and GTAp63α. **a** BN-PAGE analysis of myc-tagged TAp63α, TA*p63α, and GTAp63α. Three hundred nanogram expression vector carrying the p63 gene were transiently transfected in H1299 cells (12-well plate). Cells were harvested 24 h after transfection. Migration of the different oligomeric states is indicated by T (tetramer), D (dimer), and M (monomer). For all three isoforms only dimers are detectable. Western blots were performed with an anti-myc antibody (4A6, Merck). **b** Transactivation (TA) assay of TAp63α, TA*p63α and GTAp63α wt, on the p21 promotor. p53 was used as positive control. Hundred nanogram of each plasmid (pcDNA3, pGL3 and pRL-CMV) were transiently transfected in Saos-2 cells (12-well plate). Cells were harvested 24 h after transfection and assay was performed. Bars represent the mean value of the biological triplicate, error bars represent the standard deviation (SD), crosses represent the mean value of the technical replicates (**c**) SDS-PAGE followed by immunoblotting for myc-tagged TAp63α, TA*p63α, GTAp63α, and p53 protein levels of the TA assay performed on p21 promotor in Saos-2 cells (Fig. 2b). GAPDH was used as loading control. **d**, **e** TA titration assay of TA*p63α and GTAp63α in comparison to TAp63α on the p21 promotor. Hundred nanogram of pGL3 and pRL-CMV and increasing amount of p63 DNA (10 ng, 25 ng, 50 ng, 100 ng, 150 ng) were transiently transfected in Saos-2 cells. Empty vector was added to a total amount of 350 ng DNA for transfection (12-well plate). Cells were harvested 24 h after transfection and assay was performed. Protein levels were determined by western blotting. Fold induction is indicated with white bars, relative protein level with blue colored bars. The protein level for the highest DNA amount of TAp63α was set to 1. Dots represent protein level of each biological triplicate. **f**, **g** SDS-PAGE followed by western blotting for myc-tagged TAp63α, TA*p63α and GTAp63α protein level in the TA titration assay performed on the p21 promotor in Saos-2 cells (Fig. 2c, d). GAPDH was used as loading control

In earlier studies we had shown that inhibition of the transcriptional activity of TAp63α is mechanistically based on the formation of a closed and only dimeric state with decreased affinity to both the DNA^6,8^ and coactivators such as p300^16^. To investigate if TA*p63α and GTAp63α adopt a closed, dimeric conformation as well, we expressed both isoforms and TAp63α as control in H1299 cells and analyzed their oligomeric state by Blue-Native (BN)-PAGE. Figure 2a shows that all three forms are present as closed dimers. Size exclusion chromatography (SEC) analysis confirmed this finding (Supplementary Fig. S1). These results suggest that the transcriptional activity of TA*p63α and GTAp63α is regulated via their oligomeric state similarly to TAp63α.

Cellular TA assays in Saos-2 and H1299 cells with overexpressed proteins show that the activity of TA*p63α and GTAp63α is even lower than the activity of TAp63α (ref. ^2^, Fig. 2b, c and Supplementary Fig. S2a–f). As a control experiment, we verified that TA*p63α and GTAp63α are both localized in the nucleus to exclude the hypothesis that the low transcriptional activity is caused by changes in the cellular localization (Supplementary Fig. S2g). Previously we have shown that artificially high overexpression levels of TAp63α result in the formation of a small fraction of the tetrameric and active conformation resulting in measurable transcriptional activity^17^. The lower activity of the two N-terminally elongated isoforms of p63 could be the result of a higher thermodynamic stability of the closed dimeric state. To investigate this question we performed a TA titration experiment by transfecting Saos-2 cells with increasing amounts of DNA (Fig. 2d–g). For TAp63α, a strong correlation between promotor induction and protein level is observed, whereas TA*p63α and GTAp63α show no increase in activity with rising protein levels. This suggests that the N-terminal extensions contribute to an additional stabilization of the dimeric and inactive state.

**Fig. 3:**
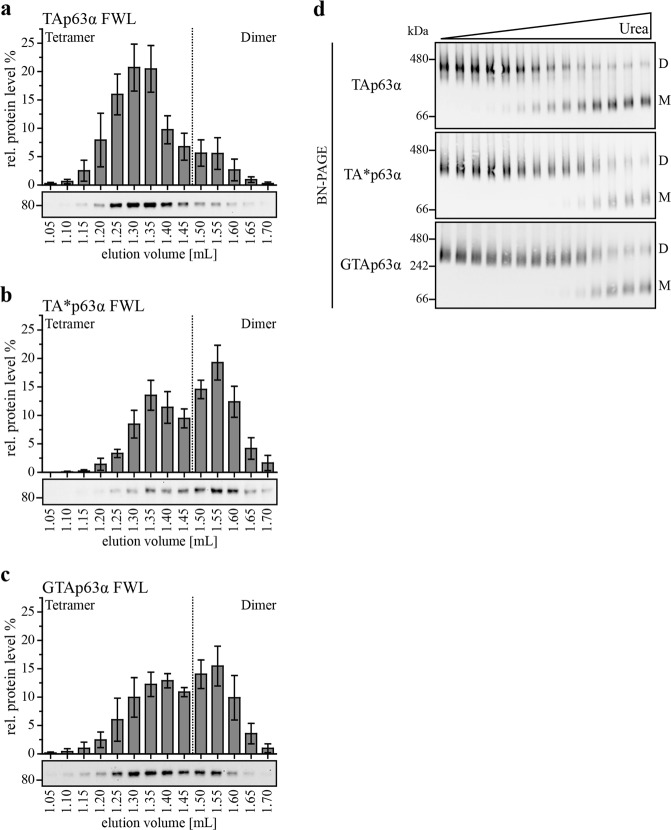
The novel isoforms show a higher kinetic stability in their dimeric state compared to TAp63α. **a**–**c** SEC analysis of TAp63α, TA*p63α and GTAp63α FWL expressed in rabbit reticulocyte lysate. Lysates were applied on a Superose 6 PC 3.2/30 column. Fractions were collected, analyzed and quantified via western blot. The sum of the intensities of all fractions corresponds to 100%. Experiments were performed five times, error bars indicate SD. **d** Urea BN-PAGE followed by western blotting for myc-tagged TAp63α, TA*p63α and GTAp63α. Two nanogram expression vector carrying the p63 gene were transiently transfected in H1299 cells (10 cm dish). Cells were harvested 24 h after transfection. Lysates were incubated with different urea concentrations on ice and applied on the gel. Migration of the different oligomeric states is indicated by D (dimer) and M (monomer). Monomers appear on the gel due to the intrinsic kinetically instability of the p63 tetramerization domain^39^

In p63, the TAD consists of a single helix^16^ that is critical for stabilization of the dimeric state^9^. Mutating the three aa F16A, W20A, and L23A (FWL mutant) in TAp63α disrupts the closed state and triggers the formation of an open tetrameric conformation^8^. We hypothesized that stabilization of the dimer by the N-terminal extensions in the TA* and GTA isoforms could result in a higher stability even in the FWL mutants. Analyzing the FWL mutant of TAp63α by SEC experiments showed the expected tetrameric state (reference^8^ and Fig. 3a). In contrast, the FWL mutants of TA*p63α and GTAp63α exhibited a significant dimeric fraction, further confirming a stabilizing effect of the N-terminal sequences (Fig. 3b, c).

The tetrameric state of TAp63α is the thermodynamically most stable one, while the dimeric state is a kinetically trapped and only metastable conformation^9^. Phosphorylation in the sequence directly preceding the TID^15^ at the C-terminus of TAp63α functions as a trigger to overcome this kinetic barrier and converts p63 into the thermodynamically preferred tetramer via a spring-loaded activation mechanism^9,12,13^. Kinetically trapped states can be converted into a thermodynamically more stable state not only by their natural trigger (phosphorylation in case of p63) but also by slight destabilization using temperature, pH, or chemical denaturants^18,19^. In earlier experiments, we have shown that relatively low concentrations of urea are sufficient to convert TAp63α into the open tetrameric state without affecting the fold of the single domains^9^. We incubated the three p63α isoforms with different urea concentrations followed by analysis on BN-PAGE. Figure 3d (and Supplementary Fig. S3) shows that for TAp63α the transition occurs at a distinctively lower urea concentration compared to TA*p63α and GTAp63α. These data clearly reveal that the inactive and dimeric conformations of TA*p63α and GTAp63α are more stable than the corresponding state of TAp63α, caused by a further stabilization by the N-terminal elongations.

### Activation of TA*p63α and GTAp63α upon chemotherapeutic agents

**Fig. 4:**
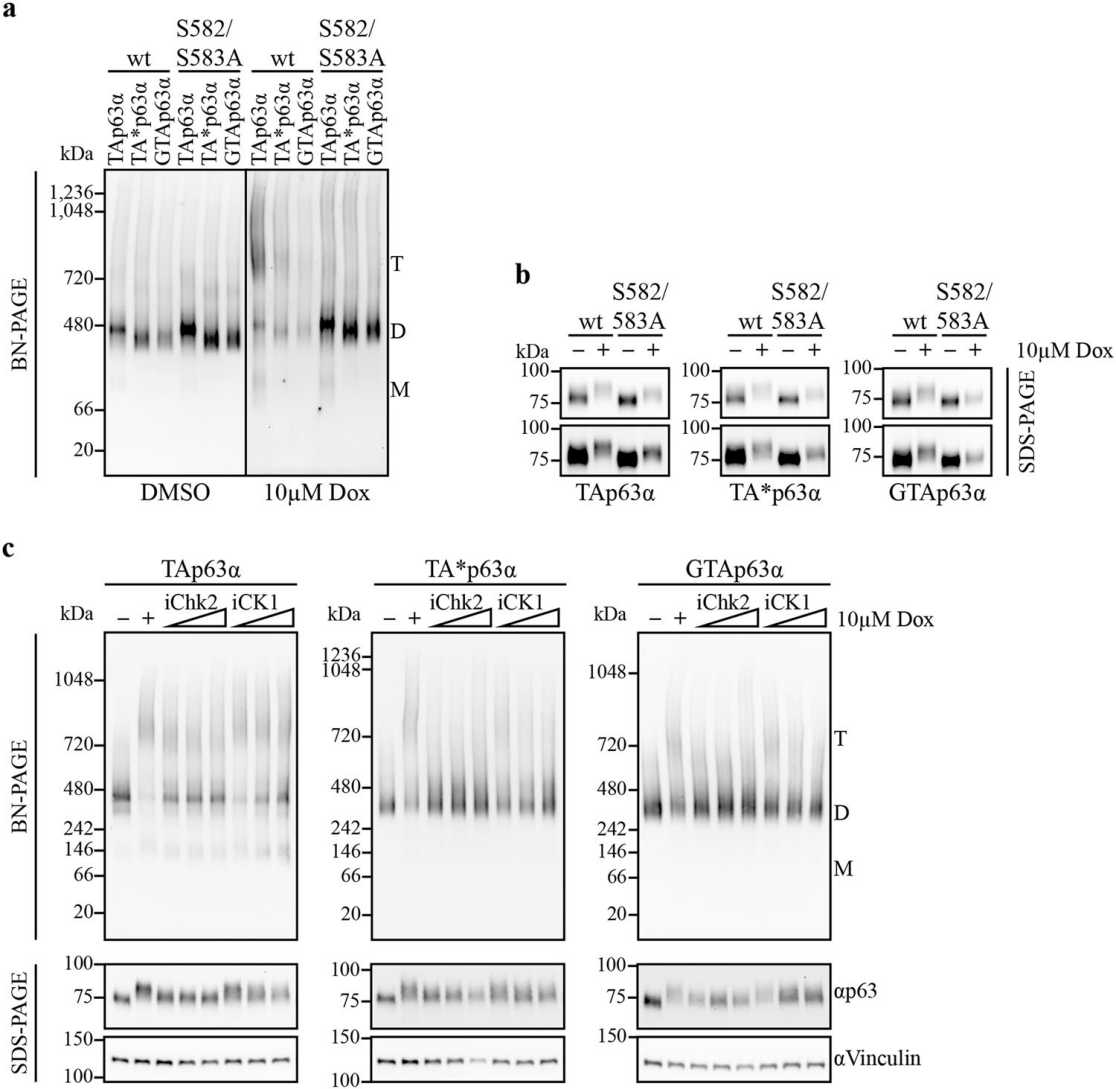
The kinases Chk2 and CK1 are able to phosphorylate and activate TA*p63α and GTAp63α like TAp63α, however, the high tetramer/dimer ratio of TAp63α cannot be reached. **a**, **b** BN-PAGE and phosphoshift SDS-PAGE analysis of wt and Chk2 priming site mutants (S582/S583A) followed by western blotting for myc-tagged TA*p63α and GTAp63α in comparison to TAp63α. Three hundred nanogram expression vector carrying the p63α gene were transiently transfected in U2OS cells (12-well plate). The next day, cells were treated with 10 µM Dox for 6 h. Migration of the different oligomeric states is indicated by T (tetramer), D (dimer), and M (monomer). The upper and lower panels in **b** show the same data with additional contrast enhancement in the lower panel. **c** BN-PAGE and phosphoshift SDS-PAGE analysis of H1299 cells lysate stably expressing either myc-tagged TAp63α, TA*p63α, or GTAp63α. Western blot analysis was carried out using an anti-myc antibody (4A6, Merck). Cells were pre-incubated with increasing Chk2 (BML-277, Merck) or CK1 inhibitor (PF670462, Sigma–Aldrich) concentrations (5, 25, 50 µM) for 1 h. Afterwards, 10 µM Dox was added and cells were harvested 6 h later. Migration of the different oligomeric states is indicated by T (tetramer), D (dimer), and M (monomer). Vinculin was used as loading control. Protein levels loaded on BN-PAGE were adjusted to equal p63 amounts by prior western blot analysis. SDS-PAGE to detect phosphoshifts were not input adjusted

In oocytes, TAp63α is activated upon DNA damage, caused by e.g., genotoxic stress, via a phosphorylation dependent mechanism including the kinases Chk2 and CK1. We wanted to test if the more stable TA*p63α and GTAp63α dimers can be activated via Chk2 and CK1 as well. To activate the DNA damage response pathway we treated transfected U2OS and H1299 cells expressing wild-type (wt) TA*p63α or GTAp63α with 10 µM doxorubicin (Dox) for 6 h. As a control, we used the S582/S583A mutants, which remove the Chk2 priming site preventing tetramerization^13^. Figure 4a, b (and Supplementary Fig. S4a, b) demonstrate that treatment with Dox activates all three isoforms to adopt tetrameric states and that the S582/583A mutations prevent this activation also for TA*p63α and GTAp63α. Tetramerization can also be prevented by treatment with the Chk2 inhibitor (BML-277, Merck) or the CK1 inhibitor (PF670462, Sigma–Aldrich), further suggesting that activation of TA*p63α and GTAp63α follows the same mechanism as TAp63α^13^ (Fig. 4c).

Despite this qualitative similarity of the activation mechanism observed for all three isoforms, quantitative differences exist. An analysis of the tetramer/dimer ratio revealed that TA*p63α and GTAp63α show a significant population of the dimeric state following treatment with Dox while TAp63α is almost completely converted into tetramers (Supplementary Fig. S4c). Measuring tetramerization kinetics showed a similar behavior. While TAp63α becomes partly tetrameric already after 1 h of treatment, TA*p63α and GTAp63α start to become tetrameric between 2 h and 4 h and do not reach the high tetramer/dimer ratio seen with TAp63α (Supplementary Fig. S4c).

### Transactivation potential of TA*p63 and GTAp63

**Fig. 5:**
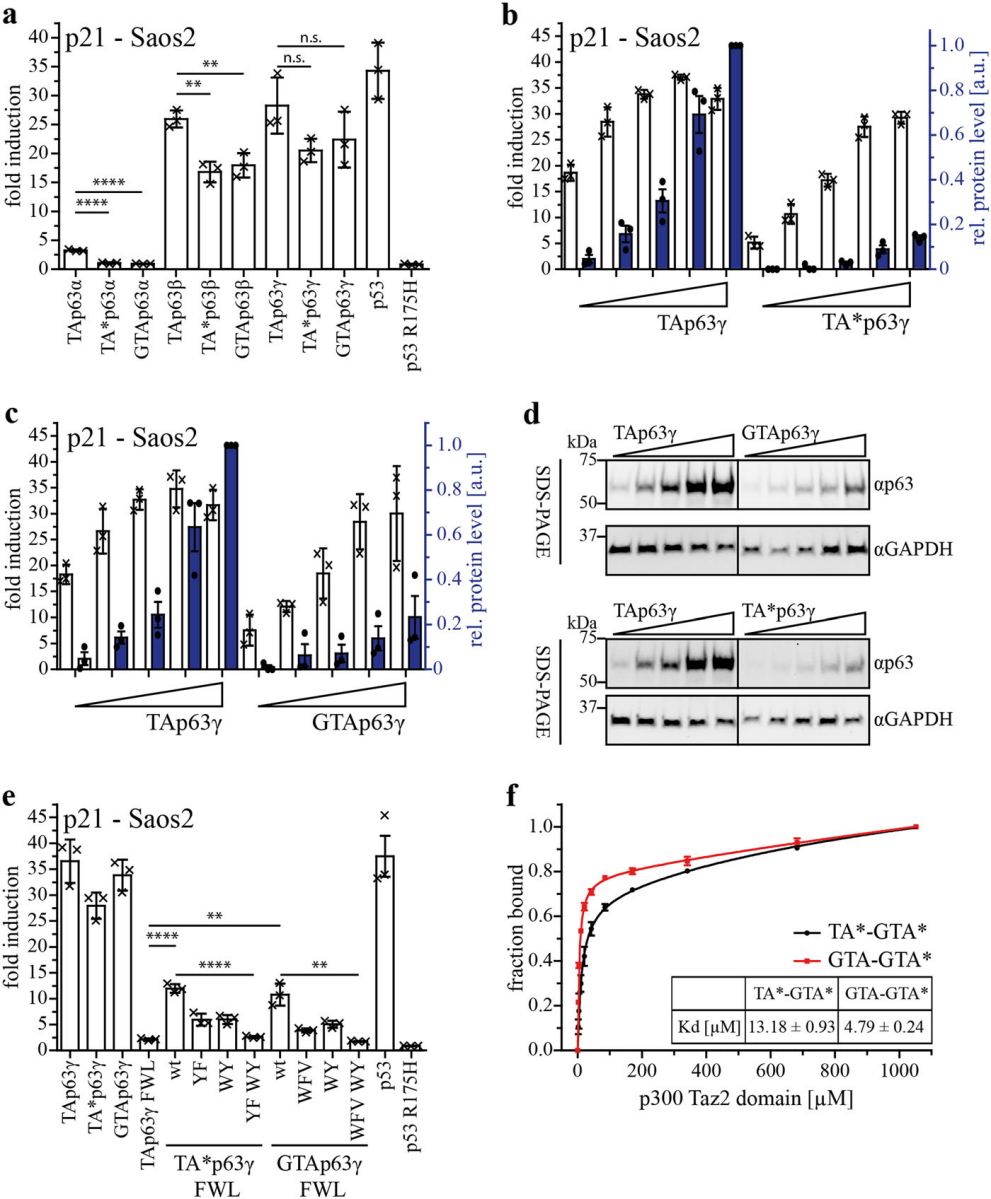
Transactivation potential of constitutively tetrameric TA*p63 and GTAp63 isoforms and its correlation with p300 Taz2 domain interaction. **a** TA assay of C-terminal TA*p63 and GTAp63 isoforms in comparison to TAp63 C-terminal isoforms on the p21 promotor. p53 wt was used as positive, the p53 R175H mutant as negative control. Hundred nanogram of each plasmid (pcDNA3, pGL3 and pRL-CMV) were transiently transfected in Saos-2 cells (12-well plate). Cells were harvested 24 h after transfection and assay was performed. **b**, **c** TA titration assay of TA*p63γ and GTAp63γ compared to TAp63γ on the p21 promotor. Hundred nanogram of pGL3 and pRL-CMV and increasing amount of p63 DNA (10 ng, 25 ng, 50 ng, 100 ng, 150 ng) were transiently transfected in Saos-2 cells. Empty vector was added to a total amount of 350 ng DNA for transfection (12-well plate). Cells were harvested 24 h after transfection and assay was performed. Protein levels were determined via western blotting. Fold induction is indicated with white bars, relative protein level with blue colored bars. The protein level of the highest DNA amount of TAp63α was set to 1. Dots represent protein level of each biological triplicate. (**d**) SDS-PAGE followed by western blotting for myc-tagged TAp63γ, TA*p63γ and GTAp63γ protein level in the TA titration assay performed on p21 promotor in Saos-2 cells. GAPDH was used as loading control. p63 isoforms were detected with the p63 antibody ab124762 (Abcam) for sensitivity reasons. **e** TA assay of FWL and potential new TA motif mutants to alanine in the N-terminus of the TA*/GTAp63 isoforms (TA*p63: Y18* F22*, W31* Y35*; GTAp63:.W9′ F12′ V15′, W29′ Y33′) on the p21 promotor. p53 wt was used as positive control, the p53 R175H mutant as negative control. Hundred nanogram of each plasmid (pcDNA3, pGL3 and pRL-CMV) was transiently transfected in Saos-2 cells (12-well plate). Cells were harvested 24 h after transfection and assay was performed. Bars represent the mean value of the biological triplicate, error bars represent SD, crosses represent mean value of the technical replicates. **f** Fluorescence anisotropy experiment with the Taz2 domain of p300 and either N-terminal fluorescein-tagged TA*-GTA*- (1*-39*) or GTA-GTA*- (1′-37′) peptide. A peptide concentration of 100 nM was used for each measuring point with increasing concentration of p300 Taz2 domain

Earlier results have implied that the TA* specific N-terminus can also partially inhibit the transcriptional activity of isoforms with the short γ-C-terminus^2^. As these isoforms are unable to form a closed dimeric state, the mechanism of inhibition must be different from that observed in TA*p63α. To address this question we investigated the influence of the N-terminal peptides on the transcriptional activity in open, constitutively tetrameric β- and γ-isoforms (Fig. 5a and Supplementary Fig. S5a–c). We could not detect a significant difference in transcriptional activity on the p21 promotor, however, noticed that the protein level of TA*p63 and GTAp63 was lower compared to TAp63 (Supplementary Fig. S5a). To further relate the promotor activity to the protein level we performed a TA titration experiment (Fig. 5b–d). For this assay, it is important to work below saturation levels, which in case of TAp63γ was unfortunately reached already at low DNA concentrations. The experiment showed that the two N-terminal isoforms were not less active than TAp63, thus, the N-terminal peptides have no inhibitory effect on constitutively tetrameric isoforms of p63. In contrast, the ratio of the fold induction and protein concentration suggests that the specific transcriptional activity of the TAp63* and GTAp63 proteins is higher than the transcriptional activity of TAp63.

Based on this result we hypothesized that in an open, tetrameric state the TA* and GTA sequences might actually enhance transcriptional activity, potentially by acting as additional transactivation domains. Secondary structure predictions using Phyre2 server^20^ indeed display helical conformations for the TA*, GTA, and GTA* peptides (Supplementary Fig. S6a). In helical wheel projections of the corresponding regions, large hydrophobic residues are located on the same site of the predicted helices similar to the FWL motif in the TAD (Supplementary Fig. S6b). For the TA* peptide, these residues are Y18* and F22*, for the GTA-peptide W9′, F12′, V15′ and for the common GTA* sequence W31* and Y35* (TA* numbering).

Further analyzing the sequences with the “9 aa-transactivation domain” prediction tool (http://www.med.muni.cz/9aaTAD/)^21^ showed matches for all three sequences fitting to most of the criteria (Supplementary Fig. S6c). To experimentally investigate these predictions we mutated the residues most likely important for transactivation to alanines and investigated their influence on the transactivation potential of TAp63γ, TA*p63γ, and GTAp63γ, each carrying the FWL mutations. As expected TAp63γ FWL is inactive due to the importance of these three aa for interaction with p300^16^, while the two other FWL mutants still show distinct induction of the promotor. Only simultaneous mutation of the additional two motifs predicted in the TA*/GTA and GTA* peptides leads to a completely inactive protein (Fig. 5e and Supplementary Fig. S6d, e).

The FWL motif in the TAD binds to the Taz2 domain of the co-activator p300 with a sub-µM K_D_ (0.196 ± 0.022 µM)^16^. We investigated the interaction of the Taz2 domain with the N-terminal peptides by fluorescence anisotropy measurements. The Taz2 domain interacts with the TA*-GTA* and GTA-GTA* peptides with K_D_ values of 13.18 ± 0.93 µM and 4.79 ± 0.24 µM, respectively (Fig. 5f). The affinity of these peptides is 75- and 25-fold lower than the affinity of the TA domain; however, the overall affinity of tetrameric p63 to p300 will be enhanced by the presence of these additionally interacting sequences.

### Expression of TA*p63α in the triple-negative epithelial breast cancer cell line Sum159

**Fig. 6:**
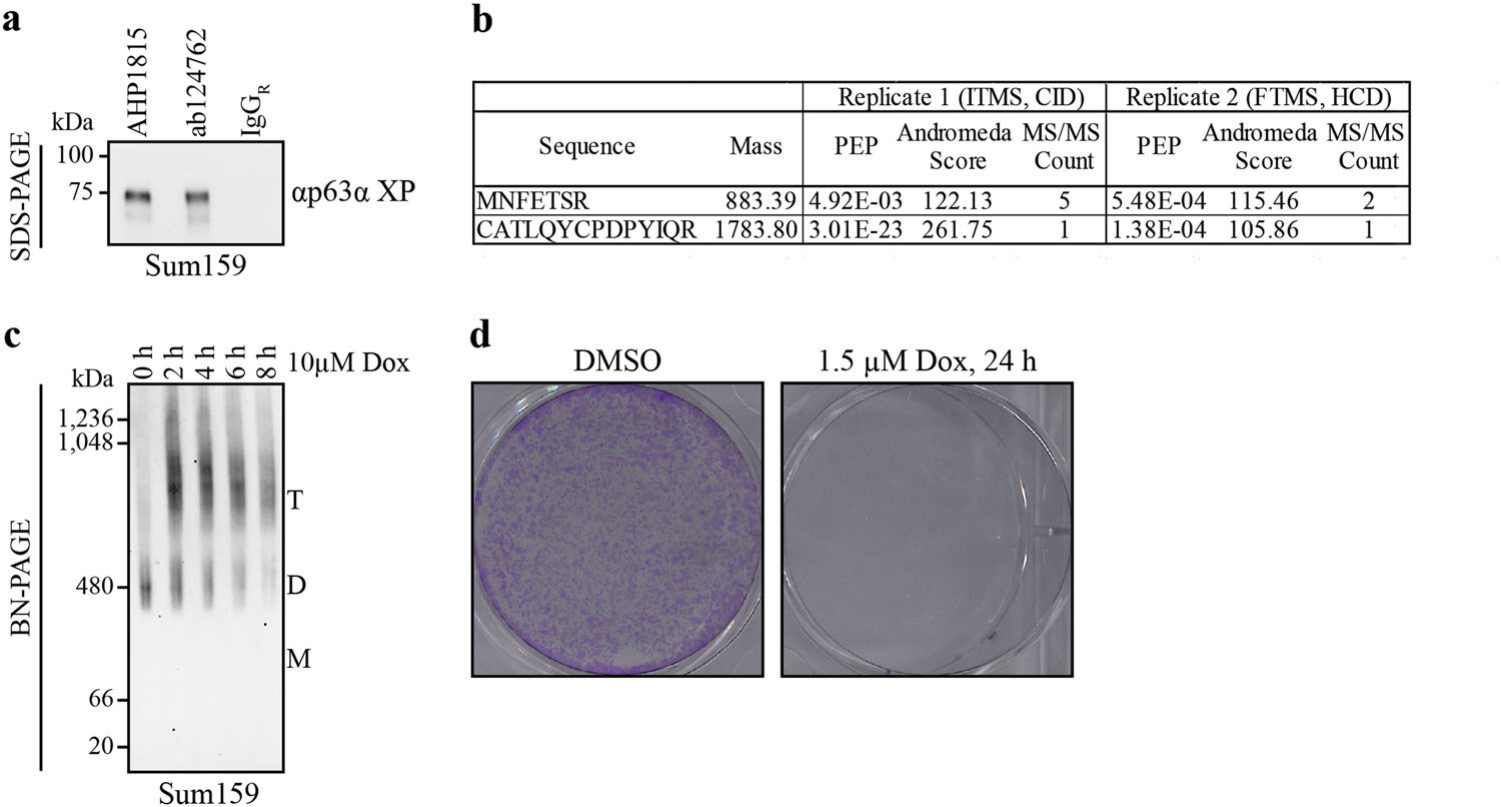
TA*p63α is expressed in the triple-negative epithelial breast cancer cell line Sum159 and activated upon Dox treatment. **a** SDS-PAGE followed by western blotting of immunoprecipitated TA*p63α in Sum159 lysate prior to MS analysis. Immunoprecipitation was performed with two different p63 specific antibodies, AHP1815 (BioRad) and ab124267 (Abcam). For detection on WB, the p63α XP (D2K8X, cell signaling) antibody was used. **b** Specific TA*-peptides were found in two independent IP-MS analyses of immunoprecipitated p63 from Sum159 cells. Immunoprecipitation was performed with the p63 antibody ab124267 (Abcam). Two different types of mass spectrometers relying on different fragmentation and detection approaches were used for the experiments (IT: ion trap, CID: collision induced dissociation, FT: Fourier transform, HCD: higher-energy collisional dissociation). **c** BN-PAGE (3–12%) of Sum159 lysate followed by western blotting for TA*p63α. Sum159 cells were treated with 10 µM Dox for different periods of time. Migration of the different oligomeric states is indicated by T (tetramer), D (dimer), and M (monomer). **d** Survival assay of Sum159 cells. Cells were treated with 1.5 µM Dox or DMSO for 24 h (six-well plate) and cultured for additional 24–30 h. Sum159 cells were fixed with glutaraldehyde (6%) and stained with crystal violet (0.5%)

p63 is rarely found mutated in tumors; however, overexpression of ΔNp63α has been reported in head and neck squamous cell carcinoma^22^ where its high expression level seems to be a driving force. Occasionally TAp63α is reported to be present in cancer cells as well. In a recent investigation of triple-negative breast cancer tissue, both ΔNp63 and TAp63 were found to be associated with different tumor characteristics^23^. TAp63α expression was correlated with androgen receptor and BRCA1/2 wild-type status and predicts a better patient survival rate. Similarly, expression of TAp63α in cervical squamous cell carcinomas showed a positive correlation with patient survival^24^. Currently no antibodies are available that could distinguish TAp63 from TA*p63. To see if cancer cells express also TA*p63 we performed immunoprecipitation (IP) experiments with lysate from the mesenchymal triple-negative breast cancer cell line Sum159. Using two different p63 specific antibodies (ab124762, Abcam; AHP1815, BioRad) for IP, we detected a band with a molecular weight of ~75 kDa on western blot (Fig. 6a). For further analysis, an IP-sample was submitted to liquid-chromatography coupled to tandem mass spectrometry (LC-MS/MS) following in-gel digestion. Two independent experiments using two different types of mass spectrometers relying on different fragmentation and detection approaches were performed. In both analyses, we could clearly identify the two N-terminal tryptic peptides of TA*p63 (MNFETSR and CATLQYCPDPYIQR) with a very low posterior error probability (PEP) and almost full fragmentation ladders (Fig. 6b, Supplementary Fig. S7) revealing that TA*p63α is indeed present in the Sum159 cell line.

To test whether TA*p63α can be activated in Sum159 cells by chemotherapeutic agents and induce apoptosis we treated cells with 10 µM Dox. TA*p63α’s oligomeric state shifted from the inactive, dimeric to the active, tetrameric form as monitored by BN-PAGE (Fig. 6c). Furthermore, the apoptosis marker cleaved PARP and cleaved Caspase3 are detectable starting 4 h following treatment (Supplementary Fig. S8). We also investigated cell survival. Sum159 cells treated with Dox (1.5 µM) or DMSO for 24 h and cultured for additional 24 h revealed a clear decrease in cell density following Dox treatment (Fig. 6d). These results suggest that TA*p63α in tumor cells could be used as a tool to fight these type of cancer cells.

## Discussion

**Fig. 7:**
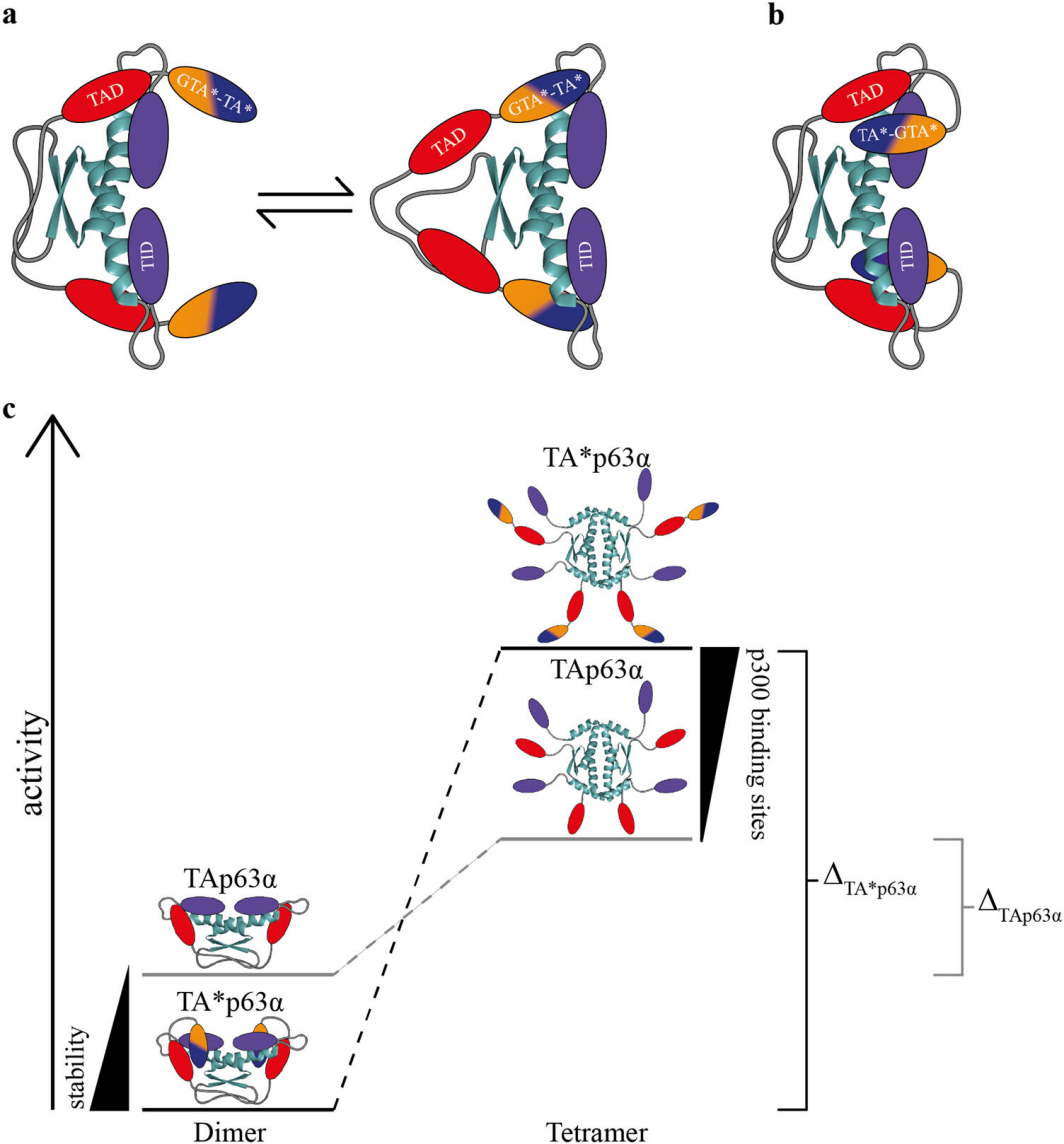
Model of the regulatory function of the N-terminal specific peptides in TA*p63α. Models are shown explicitly for TA*p63α but represent GTAp63α as well. **a** In the first model, TA*p63α’s and GTAp63α’s specific N-termini bind to the same interaction site as the TAD (red) on the TD (cyan). Consequently, the dimeric conformation has an additional “safe lock”. In case of dissociation of the TAD, the TA*-GTA* peptide (blue-orange) is able to keep the protein in its dimeric state and the TAD in close proximity for rebinding. **b** In the second model, TA*p63α’s and GTAp63α’s specific peptides are binding in their inactive, dimeric state to a further interaction site independent of the TAD keeping p63 in a more tightly packed state by building a “double lock” safety mechanism. **c** Schematic representation of the activity difference between the two oligomeric states of TA*p63α (GTAp63α) and TAp63α. The dimeric conformation of TA*p63α shows a higher stability due to the additional stabilization of the N-terminal peptides in comparison to TAp63α. The open, tetrameric state of TA*p63α, however, possess at least four further binding sites for the co-activator p300, resulting in a greater transactivation potential. Consequently, the activity difference between the inactive and active state of TA*p63α (Δ_TA*p63α_, black) is higher than the one of TAp63α (Δ_TAp63α_, gray)

The original function of the p53 protein family during evolution was probably the protection of the genetic quality in germ cells. More primitive organisms, such as *C. elegans*^25,26^ and *Nematostella vectensis*^27^, express a p53 homologue in their germs cells that based on the presence of a SAM domain is more closely related to p63 than to p53^28^. In mammals p63 gets expressed in oocytes in the diplotene phase of prophase I when cells have repaired DNA double strand breaks inflicted by Spo11 as part of the process of homologous recombination. Oocytes that have not repaired these DNA double strand breaks will be eliminated in a p63 dependent mechanism. For all remaining oocytes that have successfully repaired the DNA double strand breaks the continuously high level of p63 during the following dictyate arrest stage constitutes a constant threat. Consequently, the transcriptional activity of p63 is very tightly regulated in germ cells. Inhibition is based on blocking the tetramerization interface of the TD, which in the p53 protein family consist of a dimer of dimers^29–32^. This is achieved by a six-stranded antiparallel β-sheet that is formed by the TID of both monomers and a segment N-terminal to the DBD that adds two more β-strands per monomer^9^. The TAD of TAp63α that consists of a single helix stabilizes the closed conformation by binding to the TD as well^8^. This interaction between the TAD and the TD is the weakest structural element of the inhibitory complex due to a relatively fast kinetic off-rate of the TAD^9^. A further stabilization of the entire complex, which would result in a tighter regulation of the transcriptional activity, can therefore only be reached by modification of the N-terminal elements. Such a higher stability of the inhibitory complex is achieved by the N-terminal extensions of the TA* and GTA isoforms. There are two possible models how the N-terminal peptides stabilize the dimeric conformation. In the first, the N-terminal extension acts as an additional “safe-lock” function, binding to the same site in the TD as the TAD^8^ (Fig. 7a). In case of dissociation of the TAD from its binding site a second peptide would be in close proximity to prevent tetramerization by binding to the now vacant site on the TD and thus keeping the original TA peptide in close proximity for rebinding. In the second model, the N-terminal peptides bind to additional binding sites, building a “double-locked” dimeric conformation (Fig. 7b) and keep the protein in a more tightly packed state.

Both GTAp63α and TA*p63α can be converted into an open tetrameric form by the same two kinases as TAp63α. However, this process seems to be slower and does not reach the same level of activated tetramers compared to TAp63α. It is conceivable that effective activation of the GTAp63α and TA*p63α isoforms requires the action of an additional kinase since several potential phosphorylation sites are present in the N-termini of both isoforms. Alternatively, the N-termini of both isoforms could also (partially) occlude the kinase interaction sites. Furthermore, a faster degradation of the transcriptionally more active TA* and GTA forms could also contribute to the difference in dimer/tetramer ratio compared to TAp63α. In the case of GTAp63α, activation was shown to involve caspase-dependent cleavage of the SAM and TID^14^. A similar processing of the p63 C-termini by caspases has been suggested for TAp63α and ΔNp63α as well^33^.

In addition to their role in stabilizing the dimeric conformation the N-termini of TA*p63 and GTAp63 also support the transcriptional activity. Both N-termini have at least one further binding site for the Taz2 domain of the co-activator p300, which usually binds to the TA domain^16^. Consequently, in the active, tetrameric conformation there are at least eight interaction sites per molecule compared to four in TAp63 resulting in an increased affinity to p300 (Fig. 7c).

The question remains why the biological functions of the GTAp63α and TA*p63α require a tighter control of their transcriptional activity compared to TAp63α in oocytes. GTAp63α is expressed in mitotic spermatogonia and at decreasing levels in suprabasal, meiotic spermatocytes while haploid spermatozoa have lost expression of all p63 isoforms^14^. Spermatogonia are situated below the blood–testis barrier and comprise the germ stem cell compartment. Monitoring the genetic quality of these male germ stem cells is of critical importance to preserve germ-line integrity and ensure the evolutionary survival of a species. The very high replication activity in male germ stem cells compared with any other cell types (50–200 million spermatozoa are produced per individual per day) combined with the very long reproductive period in humanoid males might be the reason for this special type of genetic quality control. It was speculated that the incorporation of the retroviral LTR into the p63 coding sequence was a critical event that made the long reproductive period in humans possible^14^.

There are, however, distinct differences between the function of TAp63α in the female germ-line and the function of GTAp63α in male germ cells. While TAp63α gets expressed after the end of the period of homologous recombination and stays at high level throughout the cell cycle arrest phase, GTAp63α is mostly expressed in mitotically dividing stem and progenitor cells. It seems to be present also in cells undergoing homologous recombination just before meiosis. How these cells manage to suppress activation of GTAp63α triggered by the DNA double strand breaks being created is currently not known. The additional N-terminal domain might make GTAp63α more resistant to phosphorylation triggered activation. However, our experiments have shown that GTAp63α can get activated by the combined action of the Chk2 and CK1 kinases, following the mechanism seen in TAp63α in oocytes. A lack of the essential kinases or the presence of phosphatases might keep GTAp63α inactive. The time spent in the meiotic stage also clearly differs between the female and male germ cells. While oocytes are arrested in prophase of meiosis I for up to 50 years spermatocytes remain at the same stage only for hours. How these vastly differing time frames influence the genetic quality monitoring mechanisms remains to be investigated.

Our MS analysis has shown that TA*p63α is expressed in the mesenchymal triple-negative breast cancer cell line Sum159 together with a destabilizing mutant p53 form. In principle destabilized p53 mutants (such as p53R175H) can co-aggregate with p63 isoforms with an accessible TI domain^17^ and such an interaction could inactivate tetrameric TA*p63α. In preliminary experiments, we could, however, not see an interaction between both proteins in Sum159 cells so far. Several other investigations have shown that TAp63α is expressed in different cancer types^34^. Why a cancer cell expresses a pro-apoptotic wild-type member of the p53 protein family is not clear. However of all possible isoforms the N-terminal elongated TA*p63α is the one with the lowest basal transcriptional activity but can be probably used as a tool to induce apoptosis. If other cancer cells reported to express TAp63α in reality express TA*p63α has to be determined.

## Material and methods

### Cell culture

The human osteosarcoma cell lines Saos-2, U2OS and the breast cancer cell line Sum159, were cultured in DMEM medium (with glutamine, Gibco) while the non-small cell lung cancer cell line H1299 in RPMI medium 1640 (Gibco), respectively, containing 10% FBS (Capricorn Scientific) and 100 mg/ml Penicillin/Streptomycin (Gibco) at 37 °C and 5% CO_2_. Saos-2, U2OS and H1299 were obtained from ATCC. The Sum159 cell line was a gift from Frank D. McKeon (Jackson Laboratory of Genomic Medicine, Farmington, CT, USA). All cell lines were regularly tested negative for mycoplasma contamination using a PCR-based test.

### Cloning

All constructs for transient mammalian cell transfection were cloned in a pcDNA3 vector without or with an N-terminal myc-tag. The following mutations were introduced in the p63 gene: FWL (F16A, W20A, L23A; 5′GAA TTC CTC AGT CCA GAG GTT GCC CAG CAT ATC GCC GAT TTT GCC GAA CAG CCT ATA TGT TCA GTT CAG3′), YF (Y18*A,F22*A; 5′CAG TAC TGC CCT GAC CCT GCC ATC CAG CGT GCC GTA GAA ACC CCA GCT CAT TTC 3′), WY (W31*A,Y35*A/W29′A, Y33′A; 5′GAA ACC CCA GCT CAT TTC TCT GCC AAA GAA AGT GCC TAC CGA TCC ACC GCC TCC3′), YF WY (5′CAG TAC TGC CCT GAC CCT GCC ATC CAG CGT GCC GTA GAA ACC CCA GCT CAT TTC TCT GCC AAA GAA AGT GCC TAC CGA TCC ACC GCC TCC3′), WFV (W9′A, F12′A, V15′A; 5′GTT GGG AGC AGC GGG ATG CCA CAG CGG CCA CTA AGG CCG GAA AGC CCT GTT TTG TTG AG3′), WFV WY (5′GTT GGG AGC AGC GGG ATG CCA CAG CGG CCA CTA AGG CCG GAA AGC CCT GTT TTG TTG AGA CAC CAG CAC ACT TCA GTG CCA AAG AAT CAG CCT ACC GCA GCA CCG CCT C3′), S582/S583A (5′CAT CTC CTG CGG ACC CCA GCC GCC GCC TCT ACA GTC AGT GTG G3′). In case of p53, the DNA contact mutant R175H (5′CAT GAC GGA GGT TGT GAG G CAC TGC CCC CAC CAT GAG CG3′) was generated.

### Generation of H1299 cells stably expressing p63α isoforms

For generation of stably expressing H1299 cells, the PiggyBac Transposon System (System Bioscience) was used. H1299 cells were transfected in a six-well plate using the Qiagen Effectene kit according to manufacturer’s manual. Three hundred nanogram pBQM812A (PB-Cuo-MCS-IRES-GFP-EF1α-CymR-Puro Inducible cDNA Cloning and Expression Vector) containing p63α and 100 ng pB200A (PiggyBac Transposase expression vector) were used for transfection. One day after transfection, cells were reseeded in 10 cm dishes. Two days after transfection, H1299 cells were selected using puromycin (5 µg/mL) until single colonies emerge (about 10–14 days). Single colonies (four for each construct) were isolated, expanded and tested for stable inducible expression of p63 on WB and for oligomeric state on BN-PAGE. For induction, a self-prepared 10,000x Cumate (Sigma–Aldrich) solution in ≥99.8% ethanol (Carl Roth GmbH) was used. For further experiments, one clone of each construct was chosen.

### Transactivation assay

Cells were transfected in a 12-well plate with same amount per plasmid (pcDNA3, pGL3 and pRL-CMV) using the Qiagen Effectene kit according to manufacturer’s manual and grown for 24 h. The transactivation assays were performed using the Promega Dual-Glo Luciferase reporter assay kit. Cells were washed with PBS (Gibco), harvested and assayed for firefly and renilla activity in a 96-well plate in quadruplicates. The ratio of the firefly to renilla signal was determined and normalized to empty vector. The remaining sample was used for determination of expression levels via western blot using the BioRad system. Three independent experiments have been performed for each transactivation assay. For statistical analysis, *P*-values were calculated in an unpaired *t*-test using the GraphPad t-test calculator (https://www.graphpad.com/quickcalcs/ttest1/?Format = SD).

### Western blotting

4–15 or 7.5% SDS–PAGE Mini-PROTEAN TGX gels (BioRad) were blotted using the semidry Trans-Blot Turbo Transfer system (BioRad).The following antibodies were prepared in 5% milk in TBS-T and incubated overnight at 4 °C: anti-Myc (1:1000, clone 4A6, Millipore), anti-p63 (1:2500, ab124762, Abcam), anti-p63α XP (1:1000, D2K8X, Cell Signaling), anti-GAPDH (1:20,000, clone 6C5, Merck), anti-p53 (1:500, DO-1, Santa Cruz), anti-PARP (1:1000, 9542, Cell Signaling), anti-cleaved caspase3 (1:1000, 5A1E,Cell Signaling), and anti-vinculin (1:1000, clone 7F9, Santa Cruz). Secondary antibodies, used for detection, were also prepared in 5% milk in TBS-T and incubated for 1 h at RT (goat anti-mouse HRP, 1:5000, A9917, Sigma–Aldrich; goat anti-rabbit HRP, 1:2000, Jackson Immuno Research Europe Ltd., 111–035–144). Quantification of western blot signals was performed by using ImageJ.

### Blue-Native PAGE

H1299 cells were transfected using the Qiagen Effectene kit according to the manufacturer’s manual in a 12-well plate or H1299 cells stably expressing p63 isoforms were used. Cells were harvested and lysed in NP lysis buffer (20 mM Tris, 150 mM NaCl, 2 mM MgCl_2_, 20 mM CHAPS, 1 mM DTT, complete protease inhibitor cocktail (Roche), PhosSTOP (Roche) pH 7.4 or pH 8.0). Benzonase (Merck) was added and samples were lysed on ice for 1 h. BN-PAGE (3–12%, Thermo Fisher Scientific) was performed and blotted as previously described^17,35^. Protein levels loaded on BN-PAGE were adjusted to equal p63 amounts by prior western blot analysis. SDS-PAGE to detect phosphoshifts were not input adjusted.

### Urea assay Blue-Native PAGE

H1299 cells were transfected using the Qiagen Effectene kit according to the manufacturer’s manual in a 10 cm dish, grown for about 24 h, harvested and collected in NP lysis buffer without detergent (20 mM Tris, 150 mM NaCl, 2 mM MgCl_2_, 1 mM DTT, complete protease inhibitor cocktail (Roche), PhosSTOP (Roche) pH 7.4). Samples were lysed via three freeze and thaw cycles. After centrifugation, Benzonase (Merck Millipore) was added and incubated 1 h on ice. Samples were split and incubated with different urea concentration for 1 h on ice. An equal volume of NP lysis buffer was added containing the two-fold concentration of CHAPS (20 mM Tris, 150 mM NaCl, 2 mM MgCl_2_, 40 mM CHAPS, 1 mM DTT, complete protease inhibitor cocktail (Roche), PhosSTOP (Roche) pH 7.4) and incubated for about 10 min. Afterwards, 3× NP sample buffer (60% Glycerol, 15 mM Coomassie G250) was added and the sample was loaded on the gel. BN-PAGE (3–12%, Thermo Fisher Scientific) was performed and blotted as previously described^13,35^.

### Size exclusion chromatography (SEC)

N-terminal myc-tagged p63 isoforms and mutants were expressed in vitro using rabbit reticulo lysate expression system (Promega) as described previously^8^. Lysates were centrifuged at 16,100 × g for 15 min at 4 °C. Analytical SEC was performed at the Äkta purifier system in phosphate buffer (50 mM sodium phosphate pH 7.8, 100 mM NaCl) at 4 °C using a Superose 6 3.2/300 column (GE Healthcare) (injection volume 50 μL; flow rate 50 μL/min; fraction size 50 μL). The SEC fractions were collected and analyzed via western blotting.

### Staining

H1299 cells were transfected using the Qiagen Effectene kit according to the manufacturer’s manual in a 12-well plate seeded on glass slides. Twenty-four hour after transfection, cells were washed three times with PBS, fixed with 3.7% formaldehyde for 10 min and washed again three times with PBS. Cells were permeabilized by permeabilization buffer (PBS with 0.1% Triton X-100) for 15 min and blocked for 30 min (1% BSA, 0.1% Tween-20 in PBS). Anti-Myc (1:500, A190-104A, Bethyl) and anti-β-actin (1:100, clone C4, Santa Cruz) antibody were incubated overnight at 4 °C in blocking buffer. Slides were washed three times with PBSt (containing 0.1% Tween-20), incubated with secondary antibody (1:1000 in blocking buffer, donkey anti-mouse Alexa 488, donkey anti-goat Alexa 568, Thermo Fisher Scientific) for 1 h at RT in the dark and washed three times. Coverslips were mounted using Mowiol (Carl Roth GmbH) mounting medium, including DAPI (Thermo Fisher Scientific) and dried for 1–2 days. The mounting medium was prepared as described in CSH protocols (http://cshprotocols.cshlp.org/content/2006/1/pdb.rec10255). Pictures were taken using the confocal microscope Leica TCS SP5.

### Fluorescence anisotropy

The p300 Taz2 domain was expressed and purified as described before^16^. The N-terminal fluorescein labeled peptides TA*-GTA* and GTA-GTA* were ordered at Peps4LS (Heidelberg, Germany). Hundred nanomolar peptide was used for the measurements with increasing concentration of Taz2. The experiment was performed at 22 °C using a FP-6500 spectrometer (Jasco, Gross-Umstadt, Germany) and a 115F-QS cuvette (Hellma, Müllheim). The results were fitted with GraphPad Prism 5 using a two site specific binding fit.

### Immunoprecipitation

For each MS sample, 20 × 15 cm dishes of 80%-confluent breast cancer cell lines were harvested and pooled. Cells were resuspended in lysis buffer (50 mM Tris, 150 mM NaCl, 1 mM DTT, 2 mM MgCl_2_, 0.5% NP-40, complete protease inhibitor cocktail (Roche), PhosSTOP (Roche), pH 8.0). Benzonase (Merck) was added and samples were lysed for about 1½ h at 4 °C. Lysates were cleared via centrifugation (16,100 × g, 15 min, 4 °C) and supernatant was diluted with IP washing buffer (50 mM Tris, 150 mM NaCl, 0.1% Tween-20, pH 7.8). The lysate was pre-cleared with Dynabeads protein G (Thermo Fisher Scientific) for 1 h. Afterwards, the diluted lysate was split and 6 µg anti-p63 antibody (ab124762, Abcam) or rabbit gamma globulin/IgG (Thermo Fisher Scientific) was added and incubated overnight at 4 °C. The next day, Dynabeads protein G (Thermo Fisher Scientific) were added for 2–3 h at 4 °C. Beads were washed eight times, transferred into a new tube and eluted with 1× NuPAGE LDS sample buffer (Thermo Fisher Scientific) supplemented with 50 mM DTT by incubating at 70 °C for 10 min. The supernatant was transferred in to a new tube.

### Cell survival assay

Sum159 cells were seeded in a six-well plate. Two days after seeding, cells were treated either with 1.5 µM Dox or DMSO for 24 h. The medium was changed and cells were cultured for additional 24–30 h. Afterwards, cells were washed three times with PBS and the fixation and staining solution (0.5% crystal violet, 6% glutaraldehyde) was incubated 1–2 h at RT. The solution was aspirated and the fixed cells were washed about five times with H_2_O and dried for 1–2 days at RT.

### MS sample preparation

Immunoprecipated p63 of the Sum159 breast cancer cell line was applied on SDS-PAGE Mini-PROTEAN gel (BioRad) and silver stained (SilverQuest Silver Staining Kit, ThermoFisher Scientific) for MS analysis. Stained bands were isolated and destained. Proteins were reduced with 10 mM DTT for 45 min at 56 °C and alkylated with 55 mM Iodoacetamide for 30 min at RT under light-protection. After digestion with Trypsin at 37 °C overnight, tryptic peptides were extracted from the gel pieces consecutively with 30% acetonitrile (ACN) containing 3% Trifluoroacetic acid (TFA), then 80% ACN containing 0.1% TFA and finally 100% ACN for 30 min each. Extraction solutions were combined and dried by vacuum centrifugation before reconstitution in 0.1% Formic acid for LC-MS/MS analysis.

### LC-MS analyses

For the first MS experiment, peptides were analysed on an Orbitrap Elite mass-spectrometer coupled to an easy nLC II (ThermoFisher Scientific) using a 20 cm long, 75 µm ID fused-silica column, which has been packed in house with 3 µm C18 particles (ReproSil-Pur, Dr. Maisch), and kept at 45 °C using an integrated column oven (Sonation). Peptides were eluted by a non-linear gradient from 8–40% acetonitrile over 19 min and directly sprayed into the mass-spectrometer via a nanoFlex ion source (ThermoFisher Scientific) at a spray voltage of 2.3 kV. Full scan MS spectra (300–2000 m/z) were acquired at a resolution of 120,000 at *m/z* 200, a maximum injection time of 100 ms and an AGC target value of 1 × 10^6^ charges. Up to 20 most intense peptides per full scan were isolated in the ion trap using a 2 Th window and fragmented using collision induced dissociation (CID, normalized collision energy of 35). MS/MS spectra were acquired in rapid mode using a maximum injection time of 25 ms and an AGC target value of 5 × 10^3^. Ions with charge states of 1 and >6 as well as ions with unassigned charge states were not considered for fragmentation. Dynamic exclusion settings were 1 repeat count and 30 s repeat duration as well as an exclusion duration of 90 s in order to minimise repeated sequencing of already acquired precursors.

The second experiment was analysed on a Q Exactive HF coupled to an easy nLC 1200 (ThermoFisher Scientific) using a 20 cm long, 75 µm ID fused-silica column packed in house with 1.9 µm C18 particles (Reprosil pur, Dr. Maisch), and kept at 50 °C using an integrated column oven (Sonation). Peptides were eluted by a non-linear gradient from 4–24% acetonitrile over 24 min and directly sprayed into the mass-spectrometer equipped with a nanoFlex ion source (ThermoFisher Scientific). Full scan MS spectra (350–1650 *m/z*) were acquired at a resolution of 60,000 at *m/z* 200, a maximum injection time of 20 ms and an AGC target value of 3 × 10^6^ charges. Up to 10 most intense peptides per full scan were isolated using a 1.4 Th window and fragmented using higher-energy collisional dissociation (normalised collision energy of 27). MS/MS spectra were acquired with a resolution of 30,000, a maximum injection time of 110 ms and an AGC target value of 1 × 10^5^. Single charged ions, ions with a charge state above 5 and ions with unassigned charge states were not considered for fragmentation and dynamic exclusion was set to 20 s.

### Mass spectrometry data processing

MS raw data processing was performed with MaxQuant (v 1.6.5.0) applying default parameters. Acquired spectra were searched against the human reference proteome (Taxonomy ID 9606) downloaded from UniProt (21–11–2018; 94731 sequences including isoforms) and a collection of common contaminants (244 entries) using the Andromeda search engine integrated in MaxQuant^36,37^. Identifications were filtered to obtain false discovery rates (FDR) below 1% for both peptide spectrum matches (PSM; minimum length of 7 aa) and proteins using a target-decoy strategy^38^.

### Predictions—helical wheel and secondary structure

For secondary structure prediction of the N-terminus the Phyre (Protein Homology/analogy recogniction engine V 2.0, http://www.sbg.bio.ic.ac.uk/phyre2)^20^ server was used. Based on this results, a helical wheel projection was created by NetWheels (http://lbqp.unb.br/NetWheels).

## Supplementary information

Supplementary Figures
