# Supplementary material for: TA*p63 and GTAp63 achieve tighter transcriptional regulation in quality control by converting an inhibitory element into an additional transactivation domain
Source: Cell Death Dis. 2019 Sep 17;10(10):686. doi: 10.1038/s41419-019-1936-z (PMC6746776; doi:10.1038/s41419-019-1936-z)
Supplement: Supplementary file 1 — Supplementary Figures [file 41419_2019_1936_MOESM1_ESM.docx]

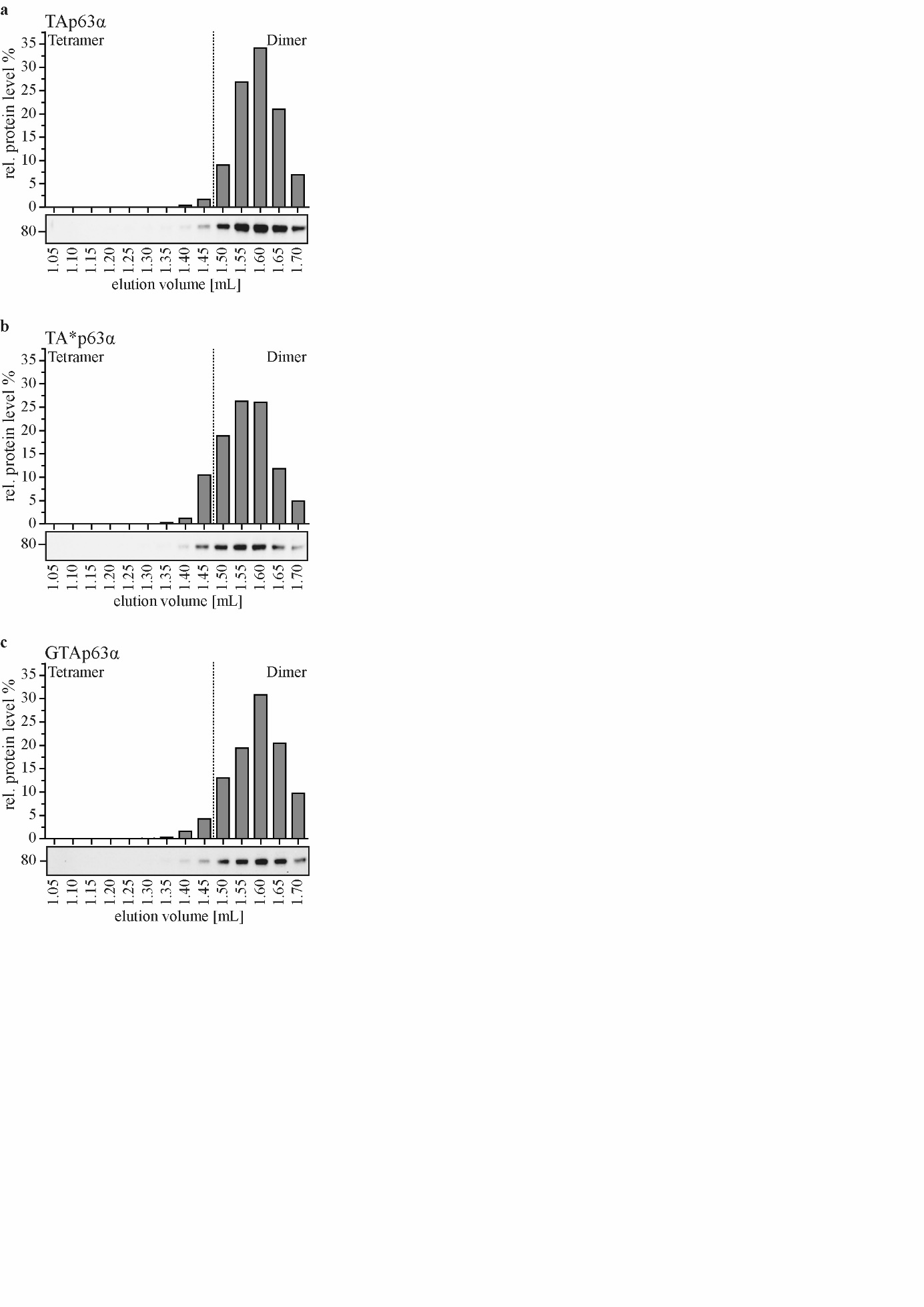


**Supplementary Figure S1**: Dimeric state of the inactive forms of TA*p63α and GTAp63α. (**a**) – (**c**) SEC analysis of myc-tagged TAp63α, TA*p63α and GTAp63α expressed in rabbit reticulocyte lysate. Lysates were applied on a Superose 6 PC 3.2/30 column. Fractions were collected, analyzed and quantified via western blot. The sum of intensity of all fractions corresponds to 100%.


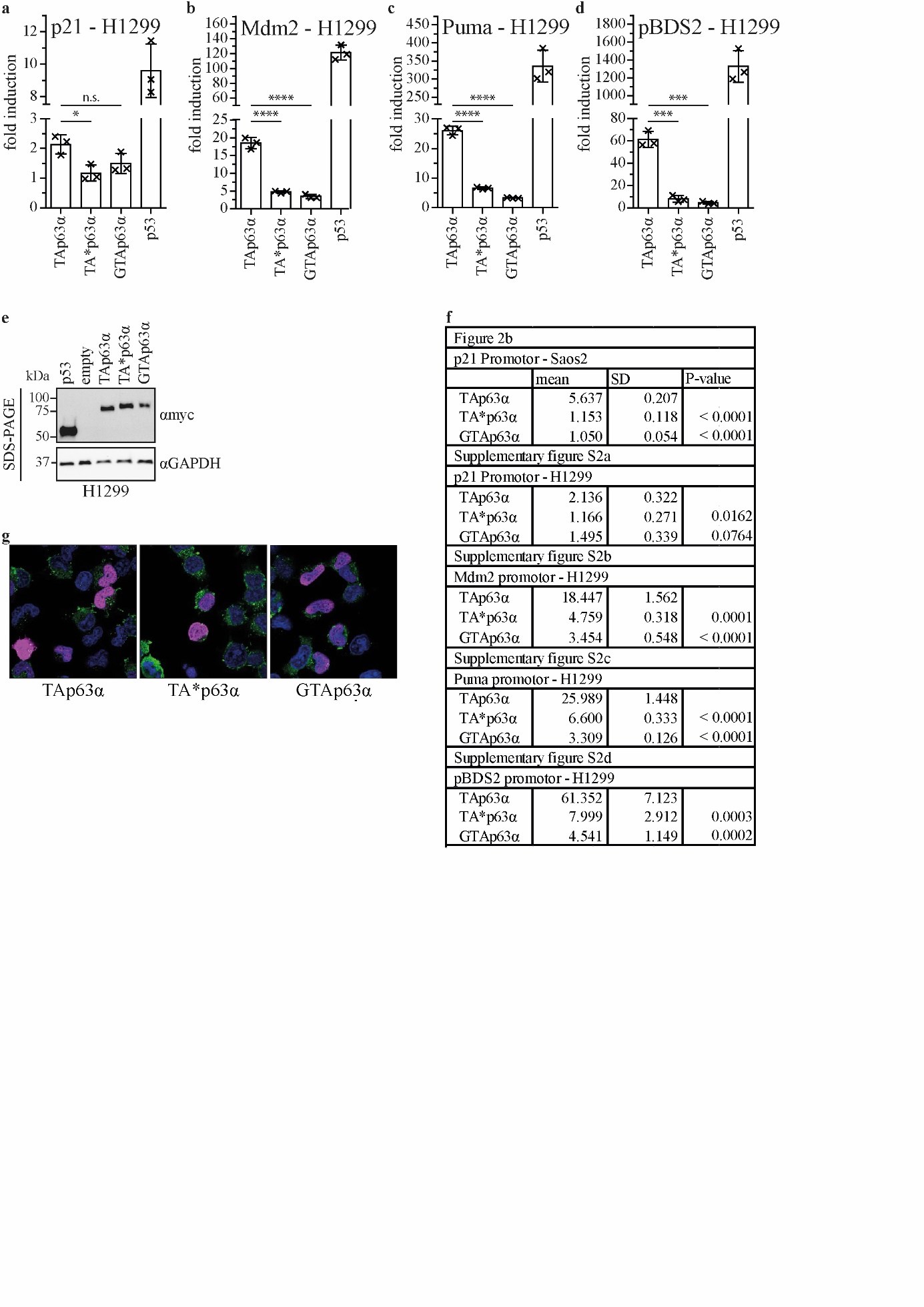


**Supplementary Figure S2:** Transactivation potential of TA*p63α and GTAp63α and their cellular localization. (**a**) – (**d**) TA assay of myc-tagged TAp63α, TA*p63α and GTAp63α on the p21, Mdm2, Puma and pBDS2 promotor (Hermeking et al., 1997, Mol Cell 1, 3-11). p53 was used as positive control. 100 ng of each plasmid (pcDNA3, pGL3 and pRL-CMV) were transiently transfected in H1299 cells (12-well plate). Cells were harvested 24 h after transfection and assay was performed. Bars represent the mean value of the biological triplicate, error bars represent the standard deviation (SD), crosses represent the mean value of the technical replicates (**e**) Exemplary SDS-PAGE followed by immunoblotting for myc-tagged TAp63α, TA*p63α, GTAp63α and p53 protein level of the TA assays performed in H1299 cells (Supplementary Figure S2a - d). GAPDH was used as loading control. (**f**) Mean values, standard deviations (SD) and p-values of TAp63α, TA*p63α and GTAp63α in TA assays on different promotors. P-values were calculated using student’s t-test. (**g**) Immunofluorescence staining of myc-tagged TAp63α, TA*p63α and GTAp63α transiently expressed in H1299 cells (red: p63, blue: Dapi, green: Actin).


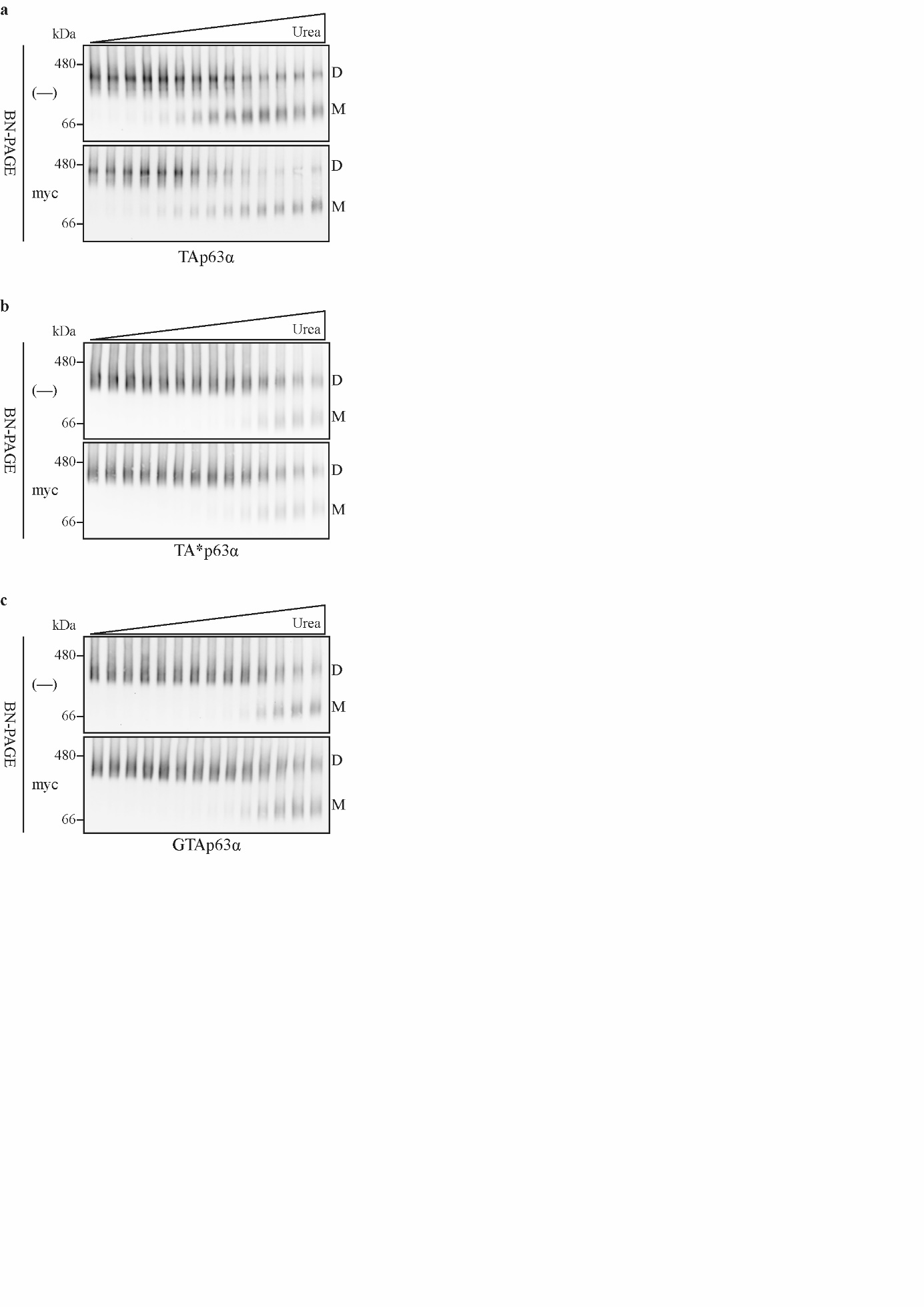


**Supplementary Figure S3:** The N-terminal myc-tag has no influence on the stability of p63α dimers. (**a**) – (**c**) Urea BN-PAGE (3-12%) followed by western blotting for untagged and myc-tagged TAp63α, TA*p63α and GTAp63α. 2 µg expression vector carrying the p63α gene were transiently transfected in H1299 cells (10 cm dish). Cells were harvested 24 h after transfection. p63 antibody ab124762 (Abcam) was used for detection. Lysate was split and incubated with different urea concentrations on ice and applied on the gel. Migration of the different oligomeric states is indicated by D (dimer) and M (monomer). As we used myc-tagged constructs, we performed this assay also with untagged p63 isoforms to exclude a potential influence of the myc-tag on the dimer stability.

**
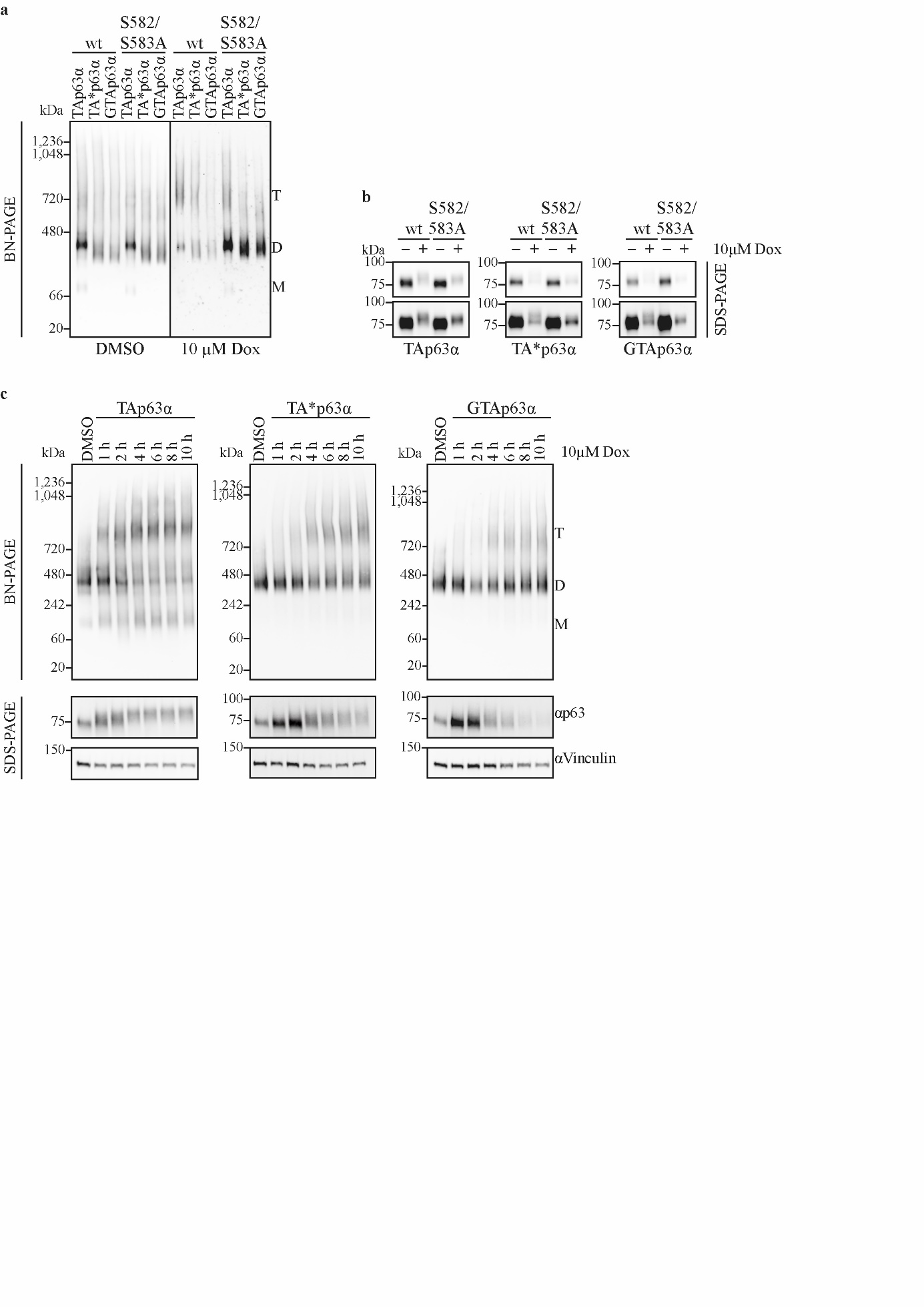
**

**Supplementary Figure S4:** The N-terminal elongated isoforms are activateable via the same mechanism as TAp63α in H1299 cells, but do not achieve predominant tetramerization like TAp63α. (**a**) + (**b**) BN-PAGE and phosphoshift SDS-PAGE analysis of wt and Chk2 priming site mutants (S582/S583A) followed by western blotting for myc-tagged TA*p63α and GTAp63α in comparison to TAp63α. 300 ng expression vector carrying the p63 gene were transiently transfected in H1299 cells (12-well plate). The next day, cells were treated with 10 µM Dox for 6 h. Migration of the different oligomeric states is indicated by T (tetramer), D (dimer) and M (monomer). The upper and lower panels in (**b**) show the same data with additional contrast enhancement in the lower panel. (**c**) BN-PAGE and phosphoshift SDS-PAGE followed by immunoblotting of Dox-treatment time line of TA*p63α and GTAp63α in comparison to TAp63α. Stably p63 expressing H1299 were treated with 10 µM Dox for up to 10 h. Protein levels loaded on BN-PAGE were adjusted to equal p63 amounts by prior western blot analysis. SDS-PAGE to detect phosphoshifts were not input adjusted.

**
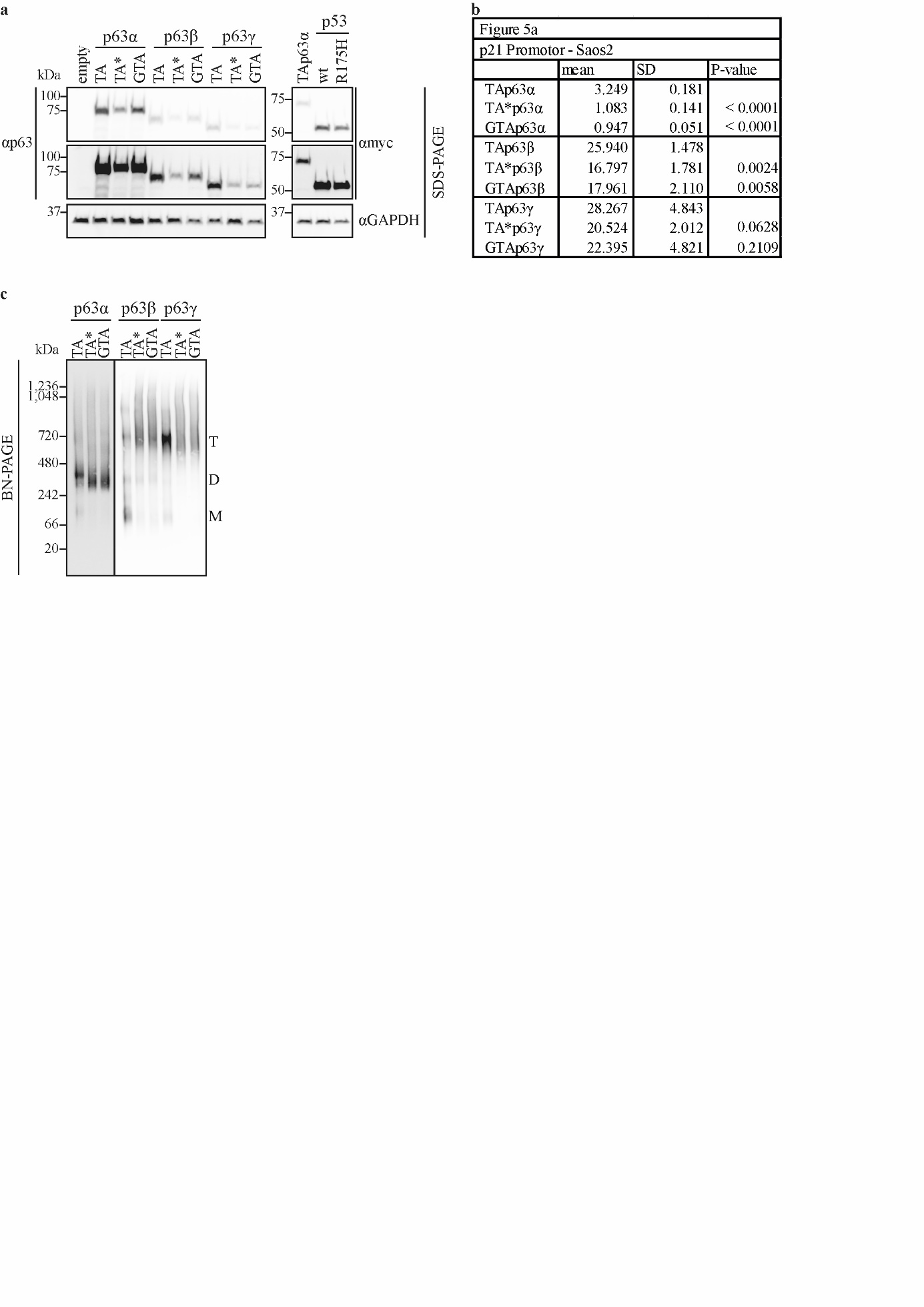
**

**Supplementary Figure S5**: Transactivation potential and oligomeric state of TA*p63 and GTAp63 isoforms. (**a**) SDS-PAGE followed by immunoblotting for myc-tagged TAp63, TA*p63 and GTAp63 C-terminal isoforms’ protein level of the TA assay performed on p21 promotor in Saos-2 cells (Figure 5a). p53 was detected via myc-antibody, while the p63 isoforms were detected with the p63 antibody ab124762 (Abcam) for sensitivity reasons. GAPDH was used as loading control. (**b**) Mean values, standard deviations (SD) and p-values of p63α, p63β and p63γ isoforms in TA assay on the p21 promotor in Saos-2 cells. P-values were calculated using student’s t-test. (**c**) BN-PAGE of p63α, p63β and p63γ followed by western blotting. 300 ng expression vector carrying the p63 gene were transiently transfected in H1299 cells (12-well plate). p63 isoforms were detected with the p63 antibody ab124762 (Abcam) for sensitivity reasons. Migration of the different oligomeric states is indicated by T (tetramer), D (dimer) and M (monomer).


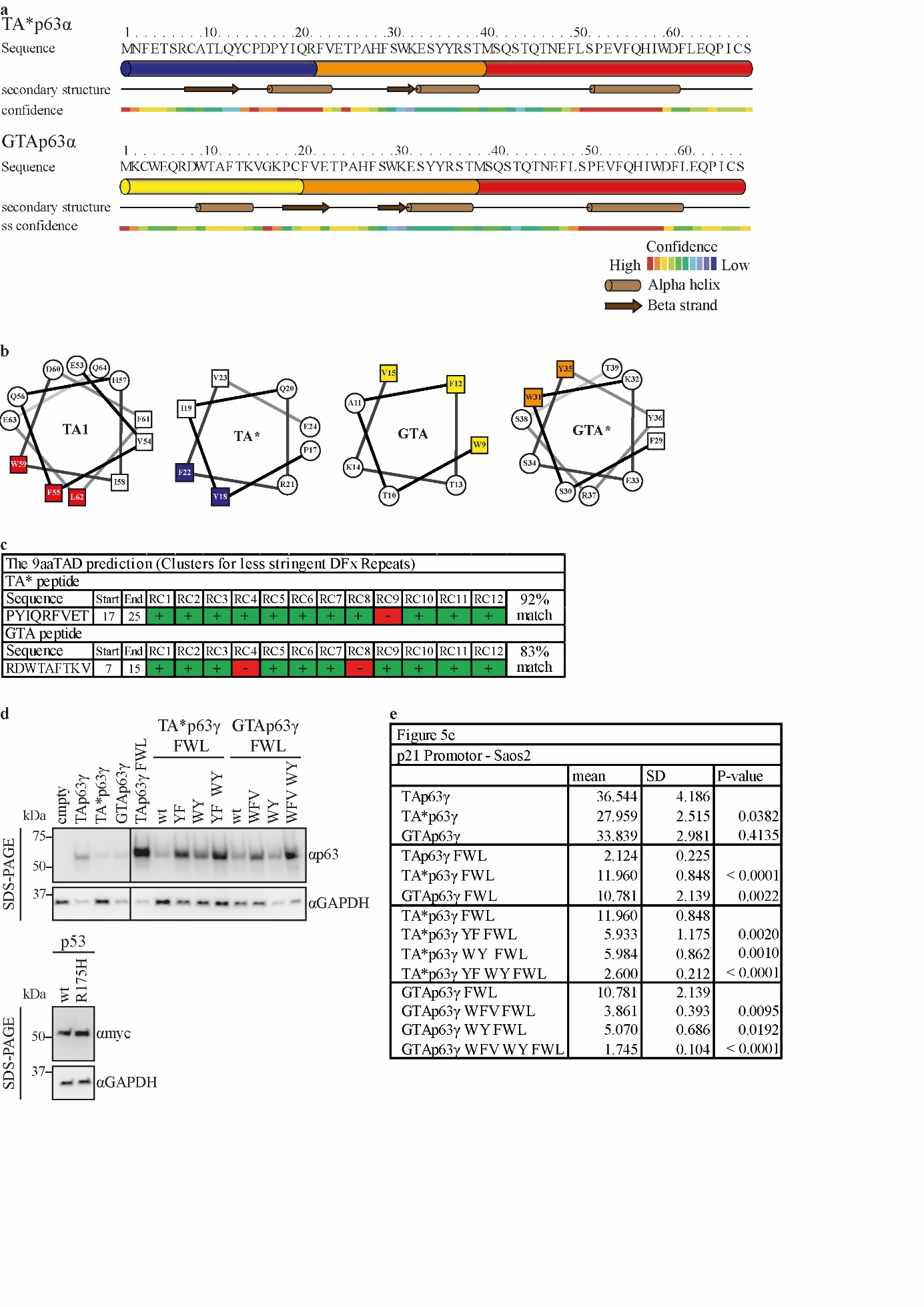


**Supplementary Figure S6:** Secondary structure and helical wheel prediction of N-terminal TA*-, GTA- and the common GTA*-peptide. (**a**) Phyre2 server was used to make secondary structure predictions of the N-terminal extensions of TA*p63α and GTAp63α (Protein Homology/analogy recogniction engine V 2.0, <http://www.sbg.bio.ic.ac.uk/phyre2>)^20^. β-sheets are shown as arrows, α-helices as barrels. Confidence of structural prediction is indicated by rainbow color code. (**b**) NetWheels server was used to create a helical wheel projection of the additional N-terminal peptides *(*http://www.lbqp.unb.br/NetWheels/). Big hydrophobic residues are located on one site of the helix, homologous to the FWL, and are colored. (**c**) Prediction results for the TA*- and GTA-peptide using 9AA TAD prediction tool (using clusters for less stringent DFx repeats, <http://www.med.muni.cz/9aaTAD/>)^21^. (**d**) SDS-PAGE followed by immunoblotting for myc-tagged TAp63γ, TA*p63γ and GTAp63γ wt and mutants’ protein level of the TA assay performed on p21 promotor in Saos-2 cells (Figure 5e). p53 was detected via myc-antibody, while the p63 isoforms were detected with the p63 antibody ab124762 (Abcam) for sensitivity reasons. GAPDH was used as loading control. (**e**) Mean values, standard deviations (SD) and p-values of p63α, p63β and p63γ isoforms in TA assay on the p21 promotor in Saos-2 cells. P-values were calculated using student’s t-test.

**Supplementary Figure S7** Detection of the N-terminal peptides of TA*p63α by Mass Spectrometry. Gel-bands migrating at the height of p63 were cut, digested with Trypsin and the resulting peptides analyzed by LC-MS/MS. Shown are the MS/MS-spectra leading to the identification of the respective peptide, including the corresponding sequence and peptide fragmentation ladders. Only b- or y-ions that were present in the spectra are annotated. (**a**) and (**c**) show spectra acquired on an Orbitrap Elite using CID as fragmentation method and an ion-trap for acquisition of fragment spectra, while (**b**) and (**d**) show spectra acquired on a Q Exactive HF, where all spectra were acquired in the Orbitrap and HCD was used for fragmentation of the peptides.


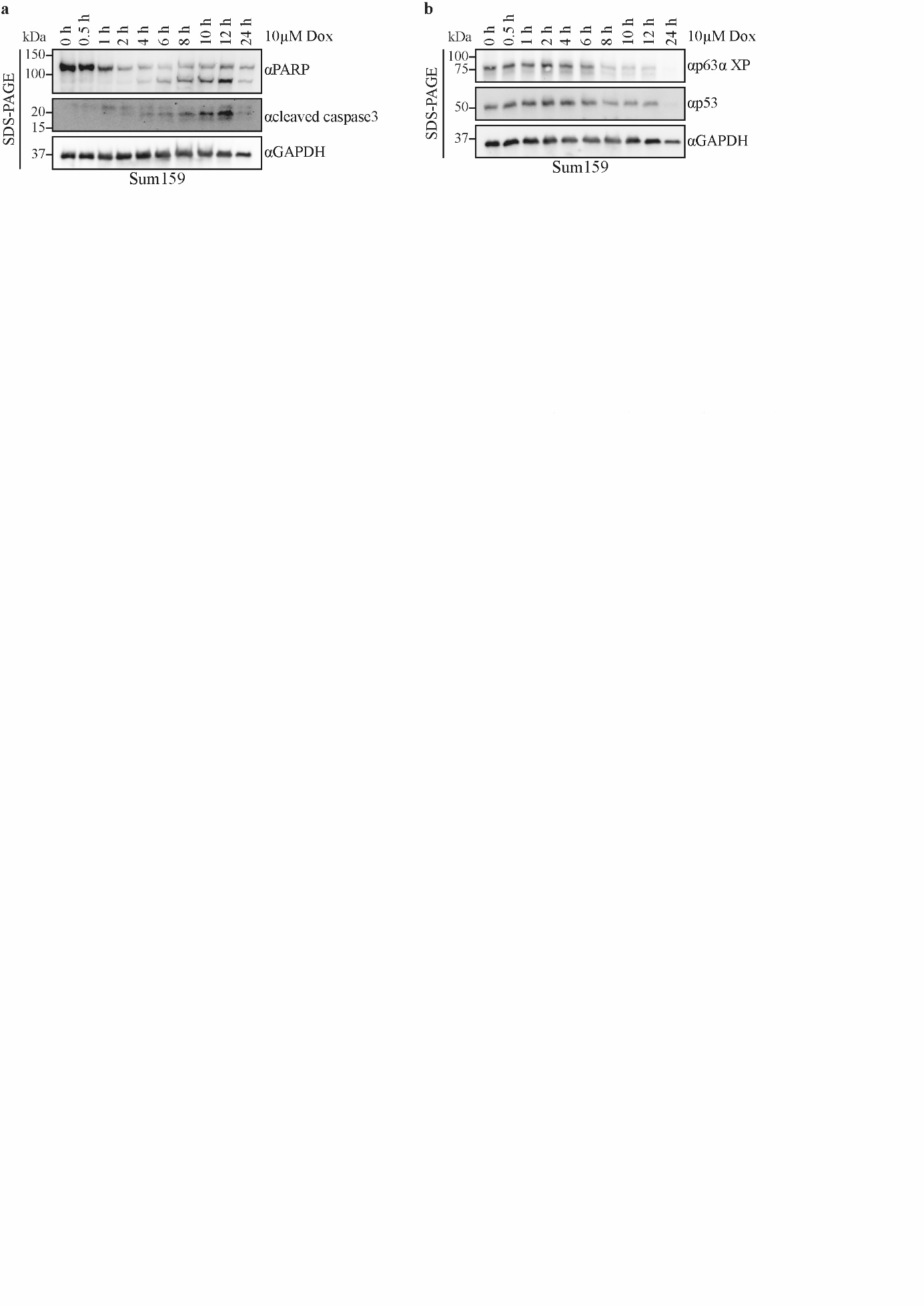


**Supplementary Figure S8** Dox treatment induces apoptosis in the breast cancer cell line Sum159. SDS-PAGEs followed by immunoblotting of Dox treated (10 µM) Sum159 cells. (**a**) Western blot was analyzed for the apoptosis markers PARP (#9542, Cell Signaling) and caspase 3 (5A1E, Cell Signaling). PARP and caspase3 cleavage were detectable starting at 4 h following treatment. (**b**) Mutant p53 (DO-1, Santa Cruz) and TA*p63α (D2K8X, Cell Signaling) levels were also investigated. A decrease in p63 and p53 level is detectable during the treatment. GAPDH was used as loading control.
